# Changes in Blood Metabolites, Intestinal Microbiota Composition and Gene Expression of 95 Weeks Old Laying Hens Differing in Egg Production and Egg Breaking Strength

**DOI:** 10.3390/ani11113012

**Published:** 2021-10-20

**Authors:** Dirkjan Schokker, Jeroen Visscher, Henri Woelders

**Affiliations:** 1 Wageningen Livestock Research, 6708 PB Wageningen, The Netherlands; henri.woelders@wur.nl; 2 Hendrix Genetics Research, Technology & Services B.V., 5831 CK Boxmeer, The Netherlands; Jeroen.Visscher@hendrix-genetics.com

**Keywords:** laying hens, vitality, (blood) metabolites, intestinal gene expression, intestinal microbiota

## Abstract

**Simple Summary:**

The average cycle of laying hens is prolonged by improving one or more aspects, including genetics, nutrition, and management. Yet, this prolongation needs to go hand-in-hand with laying hens staying vital. Our objective was to explore hen vitality at an age of 95 weeks in association with performance parameters (egg production and breaking strength). To this end, we measured metabolism and disease indicators in blood, microbiota composition and diversity in different gut segments, and the biological activity of the small intestine. We observed that 12% of the hens developed certain aberrations. Additionally, five metabolites were significantly associated to these aberrations, and two metabolites to the performance parameters. In the small intestine we observed that in the production groups the physical barrier function was affected, whereas in the breaking strength group the immune function was affected. Taken together, these data show that hen vitality at later ages can still be improved and we provided data on a molecular level that could be used in future endeavors to improve animal health and welfare.

**Abstract:**

Herein, we investigated to what extent molecular phenotypes of the systemic level (blood) and local (intestine) are associated with the performance of laying hens at 95 weeks of age. After the trial had run for 95 weeks, two performance groups were generated, i.e., egg production (PROD) and egg breaking strength (BS). A subset of 21 cages, 116 hens, was measured to indicate the metabolism and disease status. Additionally, a focus group (four cages) was made to perform molecular phenotyping in the intestine. A notifiable observation made during the post-mortem dissection was that approximately 12% of the birds at 95 weeks had developed certain aberrations and/or impairments (denoted as organ morbidity). At the systemic level, we observed five metabolites (γGT, triglycerides, HDL, glucose, and cholesterol) significantly associated to organ morbidity, and only two metabolites (urea and aspartate aminotransferase) to the performance phenotypes. At the local level, when comparing high PROD vs. low PROD, we observed differentially expressed genes involved in cell cycle processes and the extracellular matrix. When comparing high BS vs. low BS differentially, expressed genes were observed mainly involved in immune and cell cycle-related processes. This knowledge is crucial for developing novel strategies of keeping laying hens vital.

## 1. Introduction

The average cycle of laying hens is prolonged by improving one or more aspects, including genetics, nutrition, and management. However this prolongation needs to go hand-in-hand with laying hens staying vital because, at later stages in life (>80 weeks), laying hens have an increasing incidence to develop abnormalities and/or other impairments, which we defined as organ morbidity. A central organ involved in this vitality is the intestine, the primary functions of which include digestion of the feed, uptake of nutrients, and the monitoring of the environment/immune surveillance [1]. Next to the earlier-mentioned intestinal functions from the host’s perspective, the (resident) microbiota also has a complex interplay with the host, collectively playing an important role in the behavior of the intestinal ecosystem. Consequently, the intestine will signal on a systemic level, via specific molecules in the blood, about the intestinal (health) status.

The development of these intestinal functions [2,3,4] is the outcome of the interplay between host and microbiota in early life, where it is already been shown that the chicken gut microbiota composition changes during life [5,6]. In early life, successive changes are observed in both composition and (microbial) metabolic function(s). After this period, a more stable state is reached, often termed as “adult-like”, harboring a diverse set of microbial species. In humans, it is described that at old age (>65 years of age), there may be a decline in physiological condition. This also affects the gut microbiota, decreasing the number of beneficial species (e.g., *Bifidobacteria*) [7]. These shifts in the gut microbiota in the elderly also showed shifts in microbial metabolism [8], thus changing the (gut) system’s behavior. This loss of certain bacterial species is correlated with increased frailty. It has also been shown that health decline in humans is associated with gut microbiota composition, and that this microbiota composition is mainly shaped by the diet [9]. Thus, in humans, food and management strategies are already being developed, but contrary to humans, animal breeding could play a specific role in livestock. By selecting specific genetic lines and/or families, it is possible to increase the prolongation of laying rate of the hens or the increase of the breaking strength of the eggs. For brown laying hens, increasing age, an increasing intestinal permeability, compromised digestive function, and poor egg quality were observed [10]. However, this study focused on specific enzymes representing specific intestinal functionality, and in-depth molecular data were not presented. 

Modulation of health-related parameters in human elderly is mainly established by dietary interventions, i.e., food supplements and/or ingredients. Feed interventions, including pro- and prebiotics, have already shown a positive effect on performance and egg shell quality [11,12,13,14]. However, in laying hens, the impact of feed, management, and genetics on molecular (gut) health parameters still needs to be addressed. To our knowledge, there is no to little information about the baseline levels of biological activity on the systemic and local (intestinal) level of laying hens of approximately 95 weeks old. The objective of this study was to investigate differences in the levels of blood metabolites (21 cages, 116 hens) and intestinal functionality (4 cages, 20 hens) in relation to the layer’s performance (egg production and breaking strength of the eggs), aiming to demonstrate the related mechanisms of keeping laying hens vital.

## 2. Materials and Methods

### 2.1. Ethics Statement

In this study, biological material and tissues from hens were used that were available from a larger proprietary research by Hendrix Genetics. Hendrix Genetics was licensed for performing this animal research and to apply animal procedures for health monitoring, and for selective euthanasia of hens at the end of their productive lives by a Certified Poultry Veterinarian. No (additional) animal procedures were performed for the study reported here.

### 2.2. General Experimental Design

**Figure 1:**
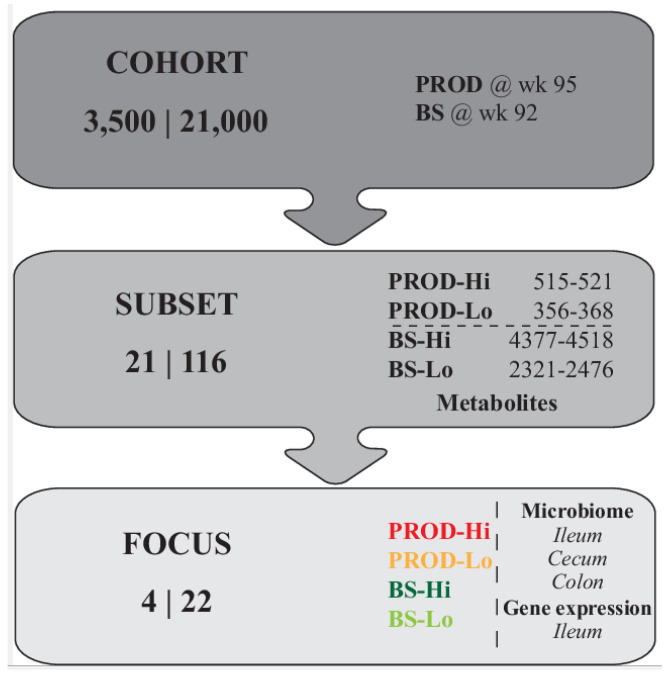
General experimental design. At the left side of each box the number of cages and hens are indicated (e.g., COHORT 3500 cages and 21,000 hens). At the right side, characteristics are shown. Top box: when the production (PROD) and breaking strength (BS) were measured. Middle box: the range of high and low PROD, PROD-Hi and PROD-Lo, respectively. As well as high and low BS, BS-Hi, and BS-Lo, respectively, metabolites were measured. The bottom box: microbiome and gene expression of specific intestinal segments that were measured.

In this study, blood metabolites, intestinal microbiota composition, and gene expression in the ileum were measured in subsets of layer hens, made available from a larger proprietary research study by Hendrix Genetics on a cohort of 21,000 hens. Hens used in the study reported here were selected from the larger cohort on the basis of recorded performance as described below (see also Figure 1). The 21,000 layers were housed in 3500 layer cages (6 birds per cage). The hens (per cage) differed in genetic background (9 combinations of 4 father lines and 4 mother lines) and feeds (four feed treatments). Hens were maintained until the age of 96 weeks and egg production (PROD, cumulative week 95) and egg Breaking Strength (BS, eggs of week 92) were monitored. Four subsets of cages were selected, comprising the 5 cages with highest BS (BS-Hi), 5 cages with lowest BS (BS-Lo), 6 cages of highest PROD (PROD-Hi), and 5 cages with lowest PROD (PROD-Lo), respectively, with a total of 116 live hens in these 21 cages at week 96. All hens were euthanized. Autopsy was performed to assess health or potential morbidity of internal organs. Blood metabolites were measured in blood from all hens of all 21 cages. Intestinal microbiota composition and gene expression were only measured in hens of one cage per subset, selected per subset on the basis of (1) control feed, (2) no hens with morbidity of internal organs, and (3) highest and lowest BS and highest and lowest PROD, respectively, with 5–6 hens per cage/subset.

### 2.3. Hen Housing

The 21,000 layers were housed at an age of approximately 17 weeks in 3500 layer cages (6 birds per cage) at a contracted research facility of Hendrix Genetics. Cages were organized in 7 stacks, 5 tiers, with controlled-random allocation of genetics/feed treatments to stacks/tiers. Hens in stacks 1, 3, 5, and 7 received control feed, whereas hens in stacks 2, 4, and 6 received feed with a feed addition, i.e., a probiotic (CloStat^®^ (Bacillus subtilis PB6, Kemin, Des Moines, IA, USA) in stack 2, a prebiotic (ButiPearl^®^, an encapsulated source of butyric acid, Kemin, Des Moines, IA, USA) in stack 4, and the a symbiotic (CloStat^®^ and ButiPearl^®^, Des Moines, IA, USA) in stack 6. Hens were maintained until the age of 96 weeks and health, hen livability and percentage lay (number of eggs laid per day per live hen), and cumulative eggs per hen housed (PROD) were monitored at flock level. PROD is the number of eggs produced per cage divided by 6 (i.e., by the number of hens per cage at start). Thus, PROD combines the fraction of hens which remained alive (livability) and production per live hen. Hens that died were removed from the cages. In week 92, a sample of 3–5 eggs for the breaking strength (BS) cages was used to assess BS. For the PROD cages, this was not determined for four cages or 1–6 eggs were used for the other PROD cages.

### 2.4. Autopsy 

The birds were first sedated and subsequently cervical dislocation was performed. Thereafter, the health status or potential morbidity of internal organs was assessed. This was followed by collection of the samples of interest, including blood, (mid-)ileum, (left)cecum, and (mid-)colon. Intestines from hens of the selected cages (see general design, four cages, 5 or 6 hens per cage) were obtained. Samples of intestinal contents of ileum, caeca, and colon were collected, snap frozen, and stored at −80 °C for later analysis of microbiota composition. Tissue samples from the (mid-)ileum were collected and snap frozen and stored at −80 °C for later analysis of gene expression. 

### 2.5. Blood Samples 

Blood from hens of the selected cages (see general design, Figure 1), 116 hens in total, was obtained and stored at −20 °C until later use. A metabolite panel was chosen a priori to cover different biological aspects, comprising of liver function (Alanine amino transferase (ALT), Alkaline phosphatase (AP), aspartate aminotransferase (AST), gamma glutamyltransferase (γGT), Glutamate dehydrogenase (GLDH), bilirubin), kidney function (creatinine and urea), and non-specific tissue damage (Lactate dehydrogenase (LDH)). Other variables included the blood lipid profile (cholesterol and triglycerides), glucose, and proteins (total protein, albumin, and globulins). In addition, hen body weight was recorded. Note that the information regarding the relevance of blood variables is mostly acquired from the human field, and reference values for some of the measured metabolites in birds were identified [15,16,17]. Analysis of the mentioned blood (serum) metabolites was performed by Laboklin N.V. (Hoensbroek, The Netherlands), using standard blood clinical chemistry methods. 

Metabolites and other hen data, i.e., body weight, were analyzed in a linear mixed model by using the lme4 package (1.1–21) in R (v3.6.1). The following model was used: y = cage number + cage number | group + Organ Morbidity, where ‘y’ represents the metabolite value(s), ‘cage number’ denotes the cage number, ‘group’ represents PROD-Hi, PROD-Lo, BS-Hi, or BS-Lo, ‘cage number | group’ denotes that group is a factor for the random term ‘cage number’, and ‘Organ Morbidity’ denotes the fixed (binary) factor for hens that either developed certain aberrations and/or impairments or not.

### 2.6. Microbiota: Intestinal Luminal Content Samples

In total, 66 samples of intestinal content from 21 chickens (caecum, ileum, and colon) were analyzed for microbiota composition via Next Generation Sequencing (NGS). Total DNA from collected samples was isolated as described by Ladirat et al. [18] with some minor adjustments; the samples were initially mixed with 250 μL lysis buffer (Agowa, Berlin, Germany), 250 μL zirconium beads (0.1 mm), and 200 μL phenol before being introduced to a Bead Beater (BioSpec Products, Bartlesville, OK, USA) for two times for 2 min.

The microbiota composition was analyzed by using mass V4 16S rRNA amplicon sequencing [19]. For 16S rRNA amplicon sequencing of the V4 hypervariable region, 100 pg of DNA was amplified as described by Kozich et al. [20] with the exception that 30 cycles were used instead of 35; applying F515/R806 primers [21], this deviation was made because at 30 cycles the quality and quantity were already sufficient. Primers included Illumina adapters and a unique 8-nt sample index sequence key [20]. To determine the amount of bacterial DNA, a quantitative polymerase chain reaction (qPCR) using primers specific for the bacterial 16S rRNA gene was applied. The amplicon libraries were pooled in equimolar amounts and purified using the QIAquick Gel Extraction Kit (QIAGEN, Hilden, Germania). Amplicon quality and size were analyzed on a Fragment Analyzer (Advanced Analytical Technologies, Inc, Ankeny, IA, USA). Paired-end sequencing of amplicons was conducted on the Illumina MiSeq platform (Illumina, Eindhoven, The Netherlands).

The MiSeq produces sequencing data which need to be processed before the data can be interpreted. The processing was performed in a series of quality control steps, analysis of unique sequences, and classification using modules implemented in the Mothur software platform [22]. In short, the following steps were followed: (1) the sequences were trimmed to remove primer sequences and low-quality sequence data, (2) overlapping paired-ends were stitched together, (3) sequences that did not fit certain quality parameters were removed, (4) sequences were grouped, based on the barcode (the sequence tag), which allows the back-translation of sequences to individual samples, and (5) sequences were taxonomically classified using the SILVA database. After the pipeline analysis, the sequence information provided insight into the microbial diversity within the samples (in general down to the level of genus). This means that, in general, no distinction between different species can be made. For example, 16S amplicon sequencing according to the conditions used does not discriminate between different *Bacillus* species but rather indicates the sequence belonging to the Bacilli group. Furthermore, the sequence information provides semi-quantitative data on the abundance of the various micro-organisms, which is subsequently used in the predictive modelling. The data were analyzed by using R (v3.6.1) using the *phyloseq* package (v1.28.0) and rarefied to a library size of 61,204. To calculate the alpha diversity, i.e., observed species and Shannon index, the function *estimate_richness* was used. To visualize the beta diversity, the function *plot_ordination* was used, i.e., Bray-Curtis dissimilarity was used to quantify the differences in species populations. 

### 2.7. Ileal Gene Expression

Total RNA was extracted from 50 to 100 mg of whole ileum tissue (approximately 1 cm). Samples were homogenized using the TissuePrep Homogenizer Omni TP TH220P in 5 mL TRIzol reagent (Life Technologies, Carlsbad, CA, USA). The homogenate was centrifuged for 5 m at 21,000× *g*, and 350 μL of supernatant was used to isolate RNA using the Direct-zol kit (Zymo Research, Irvine, CA, USA) according to instructions of the manufacturer. Quality control was performed on the BioAnalyser (Agilent Technologies, Santa Clara, CA, USA), and quantity of RNA was determined using the Tape station (Agilent 2200 tape station, Agilent technologies, Santa Clara, CA, USA). 

Labelling of RNA was carried out as recommended by Agilent Technologies using the One-Color Microarray-Based Gene Expression Analysis Low-Input Quick Amp Labelling; 200 ng of total RNA was used as input, and 600 ng of labelled cRNA was used to hybridize the chicken microarray (Agilent Technologies, Santa Clara, CA, USA). Hybridization was performed at 65 °C for 17 h with head-over-head rotation. Microarrays were washed as recommended by the manufacturer. Microarrays were scanned using the Surescan high-resolution scanner (Agilent Technologies, Santa Clara, CA, USA) at a resolution of 3 µm, 20 bits, and PMT of 100%. Feature extraction was performed using protocol 10.7.3.1 (v10.7) for 1-color gene expression.

The data were analyzed by using R (v3.6.1) by executing different packages, including LIMMA and arrayQualityMetrics [23]. The data were read in and background corrected (method = “normexp” and offset = 1) with functions from the R package LIMMA from Bioconductor [24]. Quantile normalization of the data was carried out between arrays. The duplicate probes mapping to the same gene were averaged (‘avereps’) and subsequently the lower percentile of probes were removed in a three-step procedure: (1) get the highest of the dark spots to get a base value, (2) multiply by 1.1, and (3) the gene/probe was expressed in each of the samples in the experimental condition. To test the differences between the experimental groups, i.e., high versus low group, contrasts within the LIMMA package were generated and analyzed.

## 3. Results

### 3.1. Flock Performance

**Figure 2:**
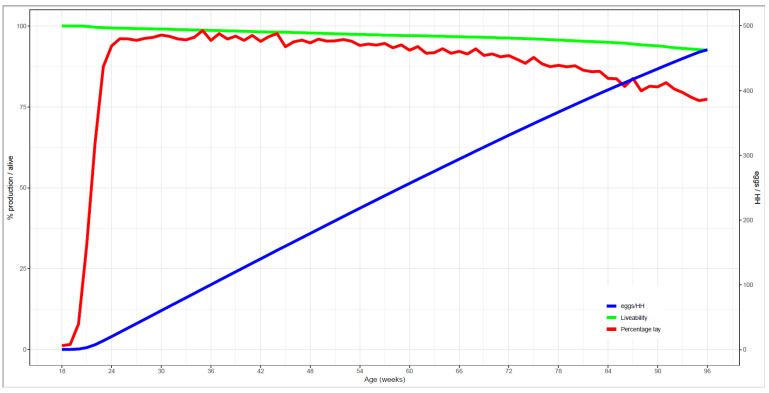
Performance statistics over the whole laying period of the flock. The percentage lay (number of eggs laid per day per live hen, red line, primary y axis), the livability (percentage hens alive, green line, primary y axis), and the eggs per hen housed (blue line, secondary y axis) are shown. The total number of weeks this flock had a percentage of lay above 90% is 49 weeks, and above 95% is 29 weeks and the flock drops below 80% of lay at 93 weeks of age.

The overall production data (Figure 2), approximately 3500 cages housing 21,000 hens, showed that most hens started laying between week 20–24 and that 50% of hens were laying in week 22. From 18 to 95 weeks of age, cage and overall flock performance was measured, i.e., percentage lay (number of eggs laid per day per live hen), livability (percentage of hens alive), and mean cumulative number of eggs laid per housed hen (Figure 2). Cumulative mortality for this flock at week 95 was 7.5%, and percentage lay was 95% at week 25, above 95% for 29 consecutive weeks, above 90% for 49 consecutive weeks (between 23 and 72 weeks of age), and below 80% at week 93. The time course of percentage lay indicated some decline of the daily egg number after week 45, followed by a stronger decline after week 75, indicating either decreased daily laying frequency of all hens, or cessation of laying of a subgroup of hens, or both. Note, the effect of feed was a part of the overall study of the cohort by Hendrix Genetics, but was disregarded in our study because the effect was negligible. In the subset of 21 selected cages in which blood metabolites were studied, many confounding factors were present; however, no confounding factors were included in the four cages, where intestinal microbiota and gene expression of the hens was studied.

### 3.2. Pathology, Blood Metabolites, and Intestinal Characteristics (in Subsets of Cages)

**Figure 3:**
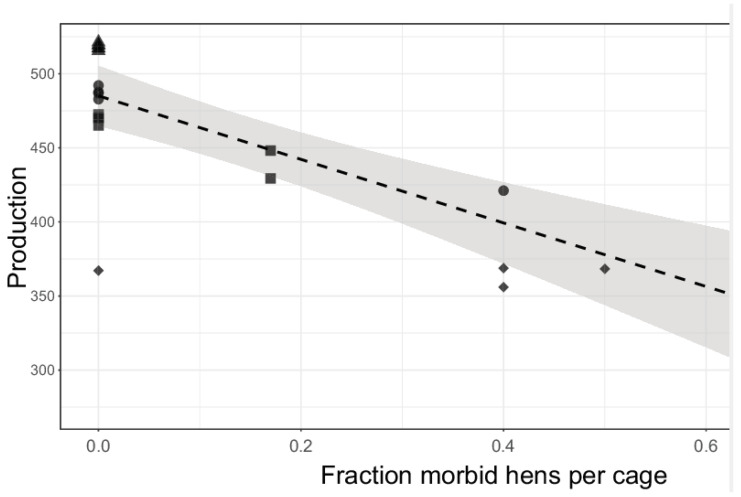
Corrected means of production per cage. The x-axis depicts the fraction of morbid hens per cage, whereas the y-axis depicts the production. The different (post hoc) groups are depicted by a triangle (PROD-hi), a diamond (PROD-Lo), a square (BS-Hi), or a circle (BS-Lo). The dotted line represents the linear regression and the gray area depicts the associated standard error.

Subsets of cages, in total 21 cages, were selected for autopsy and measurement of blood metabolites as explained above. Individual hen data (116 hens) are provided in Supplementary Table S1. Pathology (autopsy) data revealed that a fraction of the hens had severe internal organ pathology that would clearly cause cessation of egg production, including tumors (e.g., gut and oviduct), ascites (water belly), cysts in the abdomen, blockage in the oviduct, and inflamed oviduct (for more detail per hen, see Table S1). PROD of the six cages with best PROD (PROD-Hi) was 518 eggs per hen housed (range: 516–521). These six cages had no hens with recognized morbidity at week 95. Assuming that hens on average started laying in week 22, a daily egg production through week 95 would indeed correspond with 518 laying days. Figure 3 shows that PROD was correlated with the fraction of hens per cage with severe internal organ pathology, with an intercept of 485 eggs and a slope of 214. When correcting the means per cage of eggs laid per hen for the fraction per cage of hens that had severe organ pathology, the results show that eggs laid per hen for all but one cages of the subgroups would come close to the maximally attainable number of eggs produced.

### 3.3. Systemic Level: Blood Metabolites

The a priori metabolite panel encompasses diverse metabolic and/or disease markers and was analyzed by a linear mixed-effects model. The factor ‘Cage’, in which group (PROD-Hi, PROD-Lo, BS-Hi, or BS-Lo) was nested, had a significant effect on two metabolites, aspartate aminotransferase (AST), and urea. The factor organ morbidity significantly affected five metabolites, namely, γGT, triglycerides, HDL, glucose, and cholesterol (see Table 1).

When focusing on the significant metabolites for cage, the PROD-Hi group showed lower values for AST and urea compared with PROD-Lo, BS-Hi, and BS-Lo. In the three metabolites that were significant when testing for the difference in the organ morbidity group, cholesterol values were higher and glucose and triglyceride values were lower in hens that were found to have severe organ morbidity compared with the healthy subgroup (Table 2).

### 3.4. Molecular Phenotyping at the Local Intestinal Level

Intestinal microbiota and gene expression were studied in the four ‘focus’ cages that were selected on the basis of high vs. low PROD and BS, respectively (one cage per subset, see General Experimental Design). Cage performance data and individual hen body weight of the four cages are shown in Table 3. 

#### 3.4.1. Profiling Microbiota in Different Intestinal Segments

**Figure 4:**
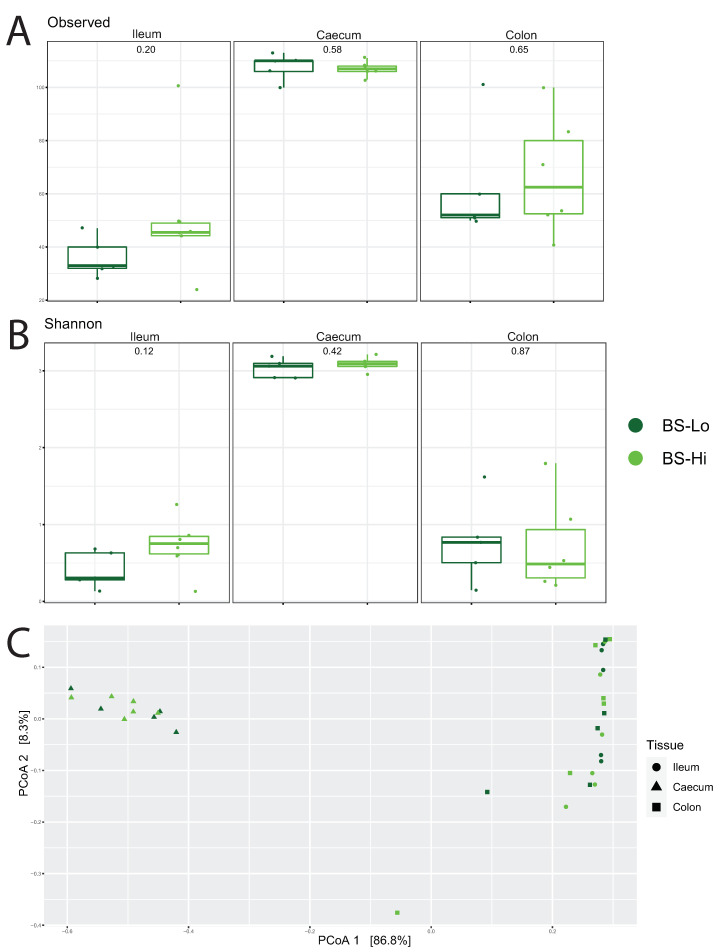
Microbiota results of various intestinal segments from hens in the high and low breaking strength. (**A**) shows the microbiota diversity, observed species, for the tissues, whereas (**B**) shows the Shannon index. (**C**) shows the microbiota composition for the ileum (circles), caecum (triangles), and colon (squares), in principal coordinate analysis (PCoA) plots. Dark green represents the low breaking-strength (BS-Lo) samples, whereas light green represents the high breaking-strength (BS-Hi) samples.

**Figure 5:**
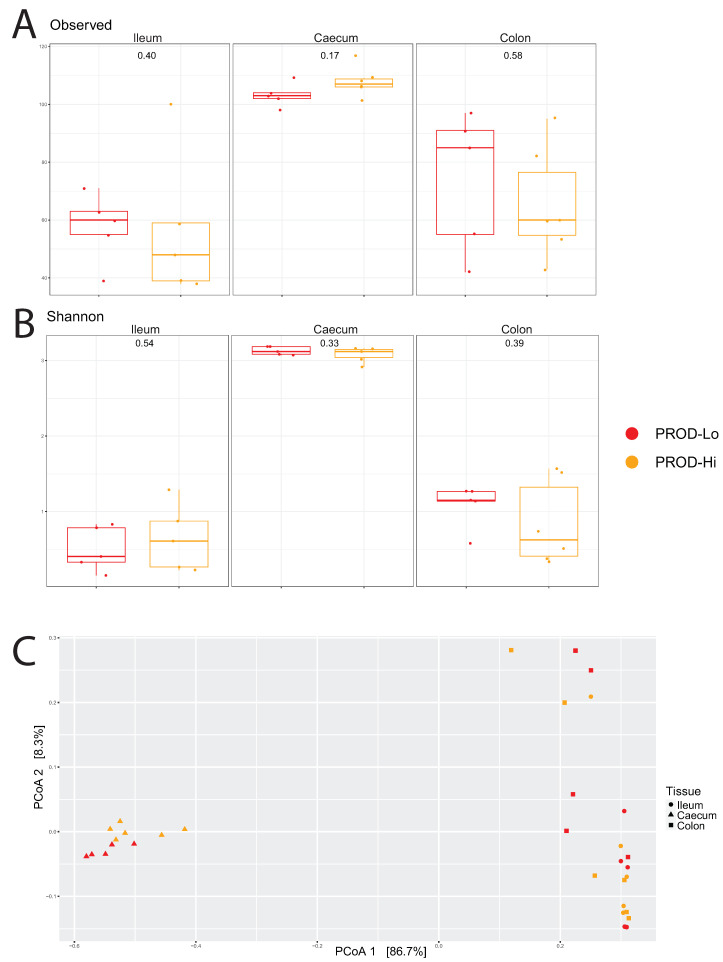
Microbiota results of various intestinal segments from the high and low production groups. (**A**) shows the microbiota diversity, observed species, for the tissues, whereas (**B**) shows the Shannon index. (**C**) shows the microbiota composition for the ileum (circles), caecum (triangles), and colon (squares), in principal coordinate analysis (PCoA) plots. Red represents the low-production (PROD-Lo) samples, whereas orange represents the high-production (PROD-Hi) samples.

In all four groups, microbiota diversity in the caeca was significantly higher than in the colon and ileum. Comparing the groups, the microbiota diversity in the caeca was only numerically higher in high-BS hens than in low-BS hens (Figure 4). The diversity values in both the ileum and colon were also numerically higher (*p*-value > 0.05) in the high-BS hens than in low-BS hens, but these differences were not significant. The principal coordinate analysis (PCoA) showed only a significant effect for tissue (*p*-value < 0.001) for both BS and PROD. Furthermore, the BS-Hi and BS-Lo hens differed slightly in caecal microbiota composition according to principal coordinate analysis (PCoA). PROD-Hi hens appeared to have lower diversity values in both the ileum and colon than PROD-Lo hens, but differences between the two PROD groups were not significant (*p*-value > 0.05, Figure 5). Additionally, PCoA did not show differences regarding microbiota composition between the two PROD groups (*p*-value > 0.05).

#### 3.4.2. Comparing the Gene Expression in the Ileum between High and Low Production and Breaking Strength Groups

Gene expression in the ileum of hens in the selected PROD-Hi and PROD-Lo cages was analyzed (as explained, one cage per subset was selected, within which hens had no morbidity and received control feed). For each individual hen, genome-wide expression data were generated, and thereafter comparisons were made between BS-Hi and BS-Lo, as well as PROD-Hi and PROD-Lo, resulting in 53 and 423 differentially expressed genes (DEGs), respectively. Table 4 shows the differentially expressed genes per contrast, specifying up- or down-regulated probes/genes.

Subsequently, DEGS (up- and down-regulated taken together) were submitted to pathway analysis. For BS-Hi vs. BS-Lo, pathway analysis resulted in scores ranging from 4.34–8.13 for the top 10 enriched pathways (Table 5), whereas for PROD-Hi vs. PROD-Lo top 10 enriched pathways, scores ranged from 10.33–14.23 (Table 6). In the enriched pathways, the maximum of hits was only 4 genes in the BS contrast and 27 genes in the PROD contrast. In the results comparing BS-Hi vs. BS-Lo, many pathways are involved in cell cycle or immune-related pathways. In the PROD-Hi vs. PROD-Lo results, many pathways relate to cell-cycle processes or the extracellular matrix. 

## 4. Discussion

This work was embedded within a larger proprietary research study by Hendrix Genetics encompassing approximately 21,000 layers in 3500 cages, in which (cumulative) egg production (PROD) and breaking strength (BS) were measured per cage. These two performance parameters are important economic traits for laying hens. At 95 weeks of age, we generated four subsets of cages with high and low PROD (cumulative week 95) and high and low BS (measured at 92 weeks), respectively. For selection of the four subsets, cages were allocated post hoc, i.e., they were not a priori groups with intrinsically different PROD or BS. Figure 3 indicates that the variation between the four subsets in the value of PROD was largely explained by the fraction of hens per cage that had severe morbidity of the internal organs. The high value of PROD at zero morbidity (485 eggs per hen for all groups; 518 in the PROD-Hi group) was close to the estimated number of 518 laying days between week 22 (start of laying) and week 95, indicating that most healthy hens laid an egg virtually on each day until week 95. The slope of Figure 3 further indicates that hens that were seen as morbid in week 95 produced on average 214 fewer eggs than the apparently healthy hens. This would be compatible with cessation of laying of these morbid hens on average around week 65. Note that the effect of genetics and feed were a part of the overall study of the cohort by Hendrix Genetics. In this cohort, many genetic families are being tested for breeding purposes, and these families could have a specific overall blood and intestinal physiological state, which may influence the results. However, feed was disregarded in our study because the effect was negligible. In the subset of 21 selected cages in which blood metabolites were studied, many confounding factors were present. However, no confounding factors, i.e., we only included control feed, were included in the four cages where intestinal microbiota and gene expression of the hens was studied.

### 4.1. Systemic Blood Metabolites in Hens with Differing Performance Parameters and Organ Morbidity

We used a linear mixed model to investigate the differences in the metabolite profiles between the four subsets. With the available small sample size of 21 cages, we could not disentangle the factor genetics (nine different combinations of 4 father and 4 mother lines) and the factor feed (four treatments), and therefore cage was the unit. For aspartate aminotransferase (AST), PROD-Hi hens had the lowest average value of 26.8, which was almost half of the AST value in the other groups, which ranged from 47.6 to 53.7. This range of AST values was close to that reported in duck [16]. Moreover, a study with one-year-old laying pheasants at the end of lay observed increasing concentrations of AST, cholesterol, phosphorus, and calcium [17]. Another study showed much higher AST levels compared with our study; however, these values of approximately 230 U/L were observed in broilers [25]. The low AST values in the PROD-Hi group (and the much higher values in the other groups) were still found when we only considered hens in which autopsy did not show morbidity. Thus, persistence of egg laying was associated with lower AST values, or rather, the cages selected for high PROD had low AST, and showed low (zero) morbidity and persistent laying until week 95. An observation similar to that for AST was made for urea, where PROD-Hi had a low average urea value (0.40) compared with the average urea values of the other groups, which ranged from 0.44 to 0.51. These findings suggest that AST can be used as an indicator for general health, i.e., absence of heart or liver damage, in laying hens.

Compared with healthy hens, morbid hens had significantly lower triglycerides and glucose, and higher cholesterol, without this being explained by the factor group (as most morbid were from PROD-Lo). Additionally, body weight (BW) was (significantly) higher in morbid hens than in healthy hens, which was only for a smaller part explained by the factor performance group (PROD or BS). In many hens, it appeared impossible to properly determine blood levels of HDL and LDL (missing data for 73 and 104 hens for HDL and LDL, respectively) because of ‘creamy’ blood. For the same reason, it was also difficult to measure bilirubin (44 missing values). Furthermore, 10 of the 14 morbid hens were seen at the high end of cholesterol values. Hens for which we had missing data for AST had on average higher cholesterol and higher creatinine. Cholesterol values varied from 1.9–10.9 mmol/L and this corresponds with earlier observations in laying hens [15]. However, here we observed that morbid hens had, on average, higher cholesterol levels compared with healthy hens. By taking all this evidence together, i.e., higher BW, higher cholesterol, we assume that the morbid hens (most of which had severe internal organ pathology that would clearly cause cessation of egg production) stopped laying and, consequently, grew fat. This is reflected by the mean BW that was 12.4% higher in morbid hens than in healthy hens, and this relationship between more abdominal fat and higher cholesterol has previously been observed as well [26]. Thus, a combination of body weight and cholesterol levels may indicate hens with possible underlying (severe) internal organ pathology.

### 4.2. Intestinal Functionality in Hens with Differing Performance Parameters

Intestinal microbiota and gene expression measurements were performed exclusively in hens from four selected cages that received control feed and of which none of the hens had organ morbidity, thus excluding additional sources of variation. Regardless of PROD and BS, i.e., in all four focus groups, actually in all individual hens, the microbiota diversity in the caeca was larger than that in the ileum and colon. This relates to the special function and anatomy of the caeca. The caeca are blind-ended sacs [27], making the passage of caecal content independent from that of the small intestine and colon. Indeed, the caecal content may rest as long as 12–20 h in the caeca, compared with a transit time of the upper intestine of only 2.5 h. A high passage time may reduce diversity as a consequence of selection on bacterial growth rate (‘wash-out’), whereas a slower passage time would allow organisms with a lower growth rate (but perhaps a better bioenergetic efficiency) to compete. Indeed, in humans, it was found that a slower intestinal transit is associated with higher bacterial diversity [28]. 

When comparing a specific segment of the GIT between individuals, it is generally assumed that a high microbiota diversity is indicative of a healthy gut function. It may be that specific bacteria species have a beneficial effect on gut health and function [29]. On the other hand, it may be that microbiota diversity is not a cause of gut health, but rather a consequence. We could, for instance, speculate that a ‘good’ healthy intestine would be characterized by a well-balanced immune response with a low level of inflammatory symptoms. In contrast, an unbalanced (too strong) immune response could negatively affect villi health and would lead to an increased passage rate, while the latter could negatively affect microbiota diversity. This negative effect has already been shown in poultry, where more aberrant and/or pathogenic microbiota populations were observed [30,31,32,33]. However, at any rate, if the microbiota diversity of individual chickens or of selected groups would indeed be a measure of gut function, this would certainly bear on the production characteristics BS and PROD, as discussed below. 

In the BS-Hi group, the microbiota diversity in the caecum was numerically higher than in BS-Lo hens. Additionally, the BS-Hi and BS-Lo slightly differed in caecal microbiota composition according to principal coordinate analysis (PCoA). In ileum mucosa, 38 genes were upregulated and 15 downregulated in BS-Hi versus BS-Lo hens, and many of these genes are involved in cell cycle or immune related processes. Upregulation of genes related to cell cycle seems consistent with larger villi with higher cell turnover. BS can depend on egg shell thickness, but also other factors can be important, such as the egg shell matrix proteins. It is known that certain genetic lines can have thicker shells containing more calcium [34,35], but with lower breaking strength than other lines. However, generally, BS is correlated with egg shell thickness [36,37], and therefore with the amount of calcium bicarbonate deposited in the egg shell. Maintaining egg shell thickness requires adequate levels of calcium in the feed, and the ability to take up the calcium. It has been suggested that the microbiota composition also may promote the intestinal absorption of calcium and support competitive exclusion of harmful bacteria [38]. Thus, it seems a likely assumption that the BS-Hi hens, which were able to maintain a high breaking strength at an age of 92 weeks, were better equipped to absorb calcium from the feed in the gut than the BS-Lo hens.

To sustain a high daily laying rate, the hen must be able to absorb nutrients (biomass and bioenergy) from the gut at a high rate. This must mean a high daily feed intake and consequently a fast (or optimal) passage through the intestine. This must certainly apply to the small intestine and colon, where steady state flux conditions must apply, and not necessarily in the caeca. The high egg production of the PROD-Hi hens would require absorption of nutrients (biomass and bioenergy) from the gut at a high rate, and, thus, a good performance of the GIT, with healthy villi and a high epithelial cell turnover. The results of gene expression of the ileum is consistent with that picture, as in these hens, 334 genes were upregulated and 89 genes were downregulated in PROD-Hi versus PROD-Lo, and many of these genes relate to cell cycle processes or the extracellular matrix. Furthermore, the high production would require a high daily feed intake and consequently a fast (or optimal) passage through the intestine. This must certainly apply to the small intestine and colon, where steady state flux conditions must apply, and not necessarily in the caeca. As discussed above, a higher passage rate could favor a decrease of microbiota diversity. The colon microbiota diversity did not differ significantly between the PROD-Hi and PROD-Lo groups. However, the numerical difference seen with numerically higher diversity mainly in the colon (and not in the caeca) in PROD-Hi hens could be a reason to look at the relation between PROD, daily feed intake, and microbiota diversity in future research. Taken together, the molecular data are only the starting point of grasping the underlying mechanisms of bird vitality in a commercial setting.

## 5. Conclusions

To our knowledge, to date, no datasets exist that describe the blood metabolite profiles, as well as intestinal microbiota and gene expression of 95-week-old laying hens. A subset of 21 cages that were post hoc selected based on their performance, i.e., cumulative egg production (PROD) and breaking strength (BS), showed differences in blood metabolites, as well as in ileum mucosal gene expression. In the post-mortem examination, we observed that 12% of the hens developed certain aberrations and/or impairments (denoted as organ morbidity). In blood, we observed five metabolites that were significantly associated to organ morbidity, including γGT, triglycerides, HDL, glucose, and cholesterol. Whereas, only two metabolites were associated to performance phenotypes, i.e., urea and aspartate aminotransferase. In ileum, we observed differentially expressed genes that were involved in cell cycle and extracellular matrix processes in PROD-Hi vs. PROD-Lo. In hens of BS-Hi vs. BS-Lo, we observed differentially expressed genes that were mainly involved in immune and cell cycle-related processes. This knowledge is crucial to understand the mechanisms of keeping laying hens vital, as the aim in the layer industry is to increase laying persistency to 100 weeks of age and beyond.

## Figures and Tables

**Table 1:** Results of the linear mixed-effects model.

Metabolite	# ^1^	*p*-Value		
		Intercept	Cage	Organ Morbidity
Globulins (g/L)	113	<0.001	0.225	0.371
γGT (U/L)	100	<0.001	0.319	0.024
LDL (mg/dL)	12	0.53	0.499	0.053
GLDH (U/L)	100	<0.001	0.096	0.577
Triglycerids (mmol/L)	113	<0.001	0.866	<0.001
Albumin (g/L)	116	<0.001	0.624	0.518
Creatinine (µmol/L)	109	<0.001	0.997	0.343
AST (U/L)	81	<0.001	0.017	0.265
ALT (U/L)	81	<0.001	0.169	0.817
HDL (mg/dL)	43	0.143	0.544	0.008
Bilirubin (µmol/L)	72	<0.001	0.722	0.299
Glucose (mmol/L)	113	<0.001	0.882	0.002
Urea (mmol/L)	111	<0.001	0.004	0.448
AP (U/L)	114	<0.001	0.226	0.068
Total Protein (g/L)	113	<0.001	0.223	0.733
Cholesterol (mmol/L)	113	<0.001	0.878	0.014
LDH (U/L)	115	<0.001	0.554	0.611

^1^ # is the number of hens per group. Note that for each metabolite, the number of hens for which we had data may be lower.

**Table 2:** Means of significant metabolites and body weight for all hens per healthy and morbid subgroup.

	n ^1^	Cage		Morbid			BW ^2^ (g)
		AST (U/L)	Urea (mmol/L)	Cholesterol (mmol/L)	Glucose (mmol/L)	Triglycerides (mmol/L)	
All hens							
*All groups*	*116*	*43.0*	*0.45*	*4.43*	*11.4*	*12.6*	*1717*
BS-hi	30	47.6	0.47	4.39	11.5	13.0	1615
BS-lo	27	47.9	0.51	4.46	11.1	12.3	1686
PROD-hi	34	26.8	0.40	4.36	11.5	13.4	1761
PROD-lo	25	53.7	0.44	4.55	11.2	11.3	1814
Healthy							
*All groups*	*102*	*41.6*	*0.44*	*4.31*	*11.5*	*13.1*	*1692*
BS-hi	28	48.1	0.47	4.41	11.6	13.2	1605
BS-lo	25	47.9	0.49	4.32	11.1	12.9	1660
PROD-hi	34	26.8	0.40	4.36	11.5	13.4	1761
PROD-lo	15	48.9	0.41	3.99	11.7	12.2	1749
Morbid							
*All groups*	*14*	*58.1*	*0.54*	*5.42*	*10.3*	*9.0*	*1901*
BS-hi	2	42.5	0.45	4.10	9.5	9.7	1754
BS-lo	2	-	0.80	6.20	10.8	5.0	2002
PROD-hi	0	-	-	-	-	-	-
PROD-lo	10	64.3	0.50	5.55	10.3	9.8	1910

^1^ Number of hens per group. Note that for each metabolite, the number of hens for which we had data may be lower. ^2^ Body weight, mean per group.

**Table 3:** Cage performance data and individual hen body weight of the four cages selected on the basis of high vs. low PROD and BS, respectively.

Subset	Cage	PROD ^1^	BS ^2^	Sample	BW (g)
PROD-hi	24.036	521	2610 (5)	55	1641
				56	1659
				57	1777.5
				58	1847
				59	1726
				60	1789
PROD-lo	22.011	367	3311 (1)	50	1772.5
				51	1620
				52	1611.5
				53	1693
				54	1733.5
BS-hi	2.053	470	4518 (3)	1	1351
				2	1593
				3	1558
				4	1559.5
				5	1447
				6	1668
BS-lo	32.074	482	2321 (3)	101	1758
				102	1731
				103	1593.5
				104	1557.5
				105	1722.5

Abbreviation used: PROD ^1^, cumulative eggs per hen housed on week 95; BS ^2^, breaking strength of the egg(s) in gram; BW, body weight.

**Table 4:** Differentially expressed genes per contrast.

Comparison	Contrast	Probes	Genes
BS-hi vs. BS-lo	Up	96	38
	Down	50	15
PROD-hi vs. PROD-lo	Up	1157	334
	Down	179	89

**Table 5:** Significantly enriched pathways ^1^ for breaking strength contrast.

Score	SuperPath Name	SuperPath Total Genes	SuperPath Matched Genes	Matched Genes (Symbols)
8.13	SMAD Signaling Network	131	3	HDAC1, ACTA1, FLNC
7.84	ICos-ICosL Pathway in T-Helper Cell	141	3	HDAC1, ITPR1, ACTA1
5.73	Proteoglycans in Cancer	242	3	ITPR1, FLNC, WNT5B
5.45	Pancreatic Secretion	100	2	ITPR1, CPA1
5.13	Immune Response Function of MEF2 in T Lymphocytes	113	2	HDAC1, ITPR1
4.74	Phospholipase-C Pathway	544	4	HDAC1, ITPR1, PLCH2, ACTA1
4.60	VEGF Pathway	138	2	ITPR1, ACTA1
4.51	NFAT and Cardiac Hypertrophy	338	3	HDAC1, ITPR1, ACTA1
4.38	FMLP Pathway	350	3	HDAC1, ITPR1, ACTA1
4.34	Adipogenesis	153	2	WNT5B, CISD1

^1^ only depicting the top 10 pathways and pathways with two or more matched genes.

**Table 6:** Significantly enriched pathways ^1^ for production contrast.

Score	SuperPath Name	SuperPath Total Genes	SuperPath Matched Genes	Matched Genes (Symbols)
14.23	Cyclins and Cell Cycle Regulation	92	9	HDAC1, RAF1, PPP2R2A, PPP2R2B, SKP1, E2F4, TP53, UBB, UBC
13.02	ADP Signalling Through P2Y Purinoceptor 12	237	14	GRK6, GNAT2, ITPR1, GNAT3, RAF1, WNT10A, CRHR2, SRC, WNT3, AKT2, UBB, UBC, ADRA2A, CLTA
13.00	ECM Proteoglycans	60	7	LUM, MATN3, NCAN, COL9A2, NCAM1, COL9A1, COMP
12.65	Peptide Ligand-binding Receptors	692	27	NMU, TRH, GNAT2, GPR6, NPY, LHCGR, GPR15, GNAT3, CCR2, PPY, PTGER2, TSPO, S1PR4, RGS14, GABRG1, PROK1, PRSS3, TACR2, WNT10A, PCDHA6, SSTR2, CRHR2, GABRB3, WNT3, SSTR4, TAS2R7, ADRA2A
11.82	Beta-catenin Independent WNT Signaling	197	12	GNAT2, ITPR1, PSMD11, PRKG2, WNT10A, SKP1, WNT5B, WNT3, CAMK2A, UBB, UBC, CLTA
11.58	Signaling By NOTCH1 PEST Domain Mutants in Cancer	118	9	HDAC1, KAT2A, FURIN, TLE3, SKP1, TP53, UBB, UBC, ATP2A1
10.83	Thyroid Hormone Signaling Pathway	127	9	HDAC1, KAT2A, RAF1, TSC2, ATP1A3, DIO3, TP53, SRC, AKT2
10.44	Ion Channel Transport	160	10	TRPV4, RAF1, TRPV6, ATP1A3, GABRB3, UBB, UBC, ATP2A1, ASIC5, BEST4
10.44	Mitotic G1-G1/S Phases	160	10	HDAC1, PSMD11, PPP2R2A, PRIM2, SKP1, E2F4, TP53, ORC6, UBB, UBC
10.33	Apoptosis Signaling Pathways	82	7	PPIG, NGFR, TNFRSF10B, TNFRSF25, PIDD1, TP53, AXIN2

^1^ only depicting the top 10 pathways and pathways with two or more matched genes.

## Data Availability

Data can be acquired on reasonable request by contacting the corresponding author.

## Supplementary Materials

The following are available online at https://www.mdpi.com/article/10.3390/ani11113012/s1, Table S1: Individual hen data.
